# Supplementary material for: Cognitive Digital Intervention for Older Patients with Parkinson’s Disease during COVID-19: A Mixed-Method Pilot Study
Source: Int J Environ Res Public Health. 2022 Nov 11;19(22):14844. doi: 10.3390/ijerph192214844 (PMC9690754; doi:10.3390/ijerph192214844)
Supplement: Supplementary file 1 [file ijerph-19-14844-s001.zip › ijerph-1998254-supplementary.pdf]

Table S1. Outcome variables at the four data collection times.

	T0	T1	T2	T3	p
MMSE score, mean±sd	27.8±1.7	27.7±2.1	28.9±1.6	28.9±1.6	0.027
ACE-R total score, mean±sd		83.6±9.7	88.4±8.1	90.2±8.2	<0.001
Attention/Orientation score, mean±sd		17.1±1.6	17.6±1.0	17.7±1.0	<0.001
Memory score, mean±sd		20.3±4.7	22.2±3.9	22.8±4.0	0.164
Fluency score, mean±sd		8.4±2.4	8.6±2.9	9.7±2.6	0.014
Language score, mean±sd		24.2±1.6	25.3±1.1	25.4±0.9	0.046
Visuo-spatial score, mean±sd		13.6±2.2	14.7±1.7	14.7±1.6	0.152
GDS, mean±sd		3.8±2.8	3.1±1.7	2.8±2.1	0.015

* p for trend calculated with repeated measures analysis of variance (ANOVA).
